# The 5′ and 3′ Untranslated Regions of the *Japanese Encephalitis Virus* (JEV): Molecular Genetics and Higher Order Structures

**DOI:** 10.3389/fmicb.2021.730045

**Published:** 2021-10-28

**Authors:** Hong Liu, Jun Zhang, Yuzhen Niu, Guodong Liang

**Affiliations:** ^1^Shandong Provincial Research Center for Bioinformatic Engineering and Technique, School of Life Sciences and Medicine, Shandong University of Technology, Zibo, China; ^2^Zibo Key Laboratory of Precise Gene Detection, Zibo, China; ^3^State Key Laboratory of Infectious Disease Prevention and Control, National Institute for Viral Disease Control and Prevention, Chinese Center for Disease Control and Prevention, Beijing, China

**Keywords:** *Japanese encephalitis virus*, *Flavivirus*, secondary structure, 5′untranslated region, 3′untranslated region

## Abstract

The untranslated region (UTRs) of viral genome are important for viral replication and immune modulation. *Japanese encephalitis virus* (JEV) is the most significant cause of epidemic encephalitis worldwide. However, little is known regarding the characterization of the JEV UTRs. Here, systematic analyses of the UTRs of JEVs isolated from a variety of hosts worldwide spanning about 80 years were made. All the important *cis*-acting elements and structures were compared with other mosquito-borne *Flaviviruses* [*West Nile virus* (WNV), *Yellow fever virus* (YFV), *Zika virus* (ZIKV), *Dengue virus* (DENV)] and annotated in detail in the UTRs of different JEV genotypes. Our findings identified the JEV-specific structure and the sequence motif with unique JEV feature. (i) The 3’ dbsHP was identified as a small hairpin located in the DB region in the 3′ UTR of JEV, with the structure highly conserved among the JEV genotypes. (ii) The spacer sequence UARs of JEV consist of four discrete spacer sequences, whereas the UARs of other mosquito-borne *Flaviviruses* are continuous sequences. In addition, repetitive elements have been discovered in the UTRs of mosquito-borne *Flaviviruses*. The lengths, locations, and numbers of the repetitive elements of JEV also differed from other *Flaviviruses* (WNV, YFV, ZIKV, DENV). A 300 nt-length region located at the beginning of the 3′ UTR exhibited significant genotypic specificity. This study lays the basis for future research on the relationships between the JEV specific structures and elements in the UTRs, and their important biological function.

## Introduction

*Flaviviruses* are enveloped viruses with 11 kb, positive-sense single-stranded RNA genome containing highly structured 5′ and 3′ untranslated region (UTRs) between which lies a single open reading frame (ORF) encoding three structural proteins (C-prM-E) and seven non-structural (NS) proteins (NS1, NS2A, NS2B, NS3, NS4A, NS4B, NS5) (Sumiyoshi et al., 1987; Liu and Qin, 2020). The NS proteins interact with certain cellular factors to form the viral replicase complex that directs the replication of the genomic RNA (Lindenbach et al., 2007; Garcia-Blanco et al., 2016; Wang et al., 2017; Sanford et al., 2019). The genome cyclizes so that a single minus strand can be synthesized with stem loop A (SLA) functioning to recruit the RNA dependent RNA polymerase (RdRp). The minus strand in the resulting double-strand serves as a template for multiple reinitiations of genome RNAs (Westaway et al., 2003; Lindenbach et al., 2007). Within the 5′ UTR, there are several conserved stem-loop (SL) structures. These include (i) SLA, which serves as a viral polymerase binding site (Filomatori et al., 2006); and (ii) Stem-loop B (SLB), which contains the upstream of AUG region (5′ UAR) (Alvarez et al., 2005) and 5′-UAR-flanking stem (UFS) (Liu et al., 2016) are involved in long-range RNA-RNA interactions and genome replication. The capsid-coding region hairpin (cHP) element lies within the coding region and aids in start codon recognition and viral replication (Clyde et al., 2008). The 5′ UAR along with 5′ downstream AUG region (DAR) (Friebe et al., 2011) and the 5′ cyclization sequence (5′ cCS) (Villordo and Gamarnik, 2009; Gebhard et al., 2011) cause genome circularization by hybridizing with their counterparts in the 3′ UTR (i.e., 3′ UAR, 3′ DAR, and 3′ cCS) (Hahn et al., 1987; Hodge et al., 2019). Besides the viral genome cyclization-related sequences, 3′ UTR has a panel of stem-loop structures that halt XRN1 exoribonuclease digestion. This results in the synthesis of sets of subgenomic flaviviral RNA (sfRNA) (Pijlman et al., 2008) which are associated with viral pathogenicity, host adaption, and immune evasion and immune evasion (Moon et al., 2012; Chapman et al., 2014; Filomatori et al., 2017).

The 5′ and 3′UTRs of the *Flavivirus* have been shown to be crucial for virus replication, immune regulation and pathogenicity (Brinton et al., 1986; Moon et al., 2012; Chang et al., 2013; Kieft et al., 2015). However, the studies of cis-acting elements in the UTRs have been mainly focused on *Dengue virus* (DENV), *Yellow fever virus* (YFV), *West Nile virus* (WNV), *Tick-borne encephalitis virus* (TBEV), and *Zika virus* (ZIKV) (Zeng et al., 2020). Although *Japanese encephalitis virus* (JEV) is arguably the most significant pathogen causing viral encephalitis within the *Flaviviruses*, few studies have been conducted to explore the functions and features of JEV UTRs. As a result, little is known about the primary and higher-order structures of JEV. Whether all of the functional important *cis*-acting elements that exist in the JEV UTRs share some features with the other *Flaviviruses* or whether JEV has some specific features has yet to be understood. It is largely unknown what the accurate locations and the sequence features of these important elements in JEV UTRs are.

*Japanese encephalitis virus* have evolved into five genetically different lineages in nature, diverged in the chronological order of GV, GIV, GIII, GII, and GI (Gao et al., 2015). JEV was initially isolated from viral encephalitis patients in 1935 in Japan and subsequently this strain was identified to be JEV GIII (Lewis et al., 1947). GIII has been the predominant genotype causing human and animal diseases in JE endemic areas until the late 20th century. In recent years, most JEV strains isolated from mosquito vectors, pigs, and humans in Asia belong to GI, thus replacing GIII as the dominant genotype in the Asian continent (Pan et al., 2011). GII primarily circulates in southeast Asia and northern Australia (Williams et al., 2000; Schuh et al., 2010), whereas GIV and GV are mainly confined to the tropical regions of Southeast Asia (Li et al., 2011; Kuwata et al., 2020; Woo et al., 2020). Both the inactivated vaccine (Nakayama) and the attenuated live vaccine (SA14-14-2) currently used for JE vaccination are derived from GIII (Ni et al., 1994; Halstead and Jacobsen, 2008; Erlanger et al., 2009). However, a growing number of reports suggest that these vaccines do not provide complete protection against GI and GV virus strains (Cao et al., 2016; Hegde and Gore, 2017). Collectively, these reports suggest that viral evolution, transmission ability, pathogenicity, and immunogenicity differ among JEV genotypes. Previous JEV studies were mainly focused on the protein-coding sequences, neglecting the study of the UTRs region. The differences in the UTRs among the JEV genotypes are poorly understood. In this study, a comprehensive study of 160 JEV strains including 67 isolates sequenced in our lab was conducted on the JEV UTRs. The results will assist our understanding of the role of UTRs in the transcription, replication and pathogenicity of JEV.

## Materials and Methods

### The *Japanese Encephalitis Virus* Untranslated Regions Data Analysis Pipeline

**FIGURE 1:**
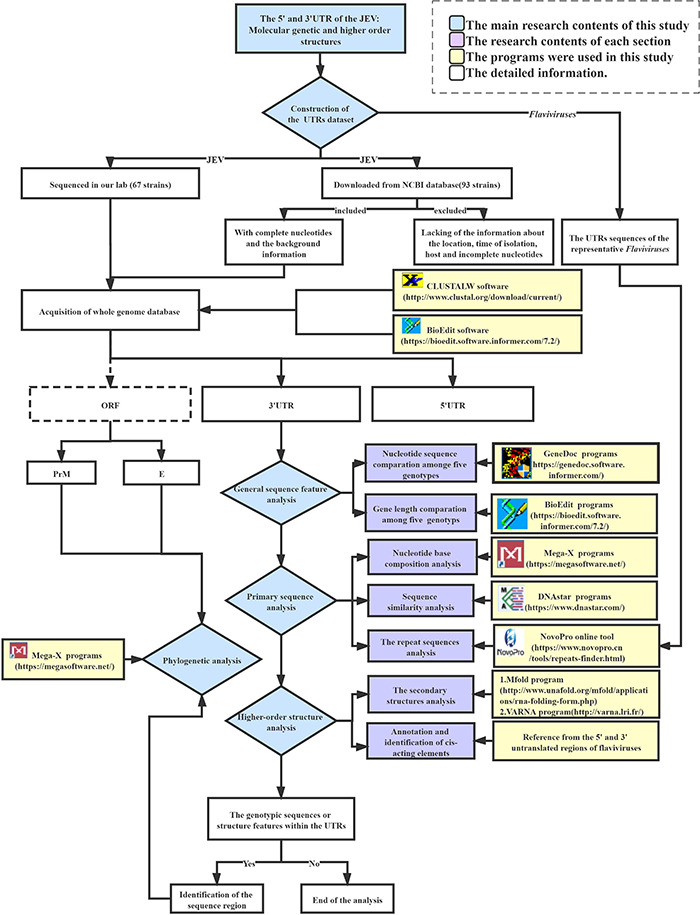
Flow chart of the JEV UTRs data analysis pipeline.

The following analyses were carried out in this work to investigate the primary sequence and secondary structural similarity and differences between representative *Flaviviruses* (WNV, YFV, ZIKV, DENV) and within the five genotypes of JEV UTRs. First, the JEV whole-genome sequence dataset was constructed, including 67 strains sequenced in our lab and 97 strains obtained from the NCBI database. Sequence inclusion criteria are described in the dataset construction section. The whole JEV genome dataset was then split into three parts to form the 5′UTR, ORF, and 3′UTR gene datasets. Meanwhile, the UTRs sequences of the representative *Flaviviruses* (WNV, YFV, ZIKV, DENV) were also downloaded as reference sequences for cis-acting element annotation and repeat sequences analysis. The general sequence feature analysis, primary sequence analysis, and higher-level structure analysis were performed using the JEV UTRs sequence datasets. The primary sequence analysis entailed sequence similarity, nucleotide composition, and repeat sequence analysis. The higher-level structural analysis comprised of the secondary structure and *cis*-acting elements annotation. Through these analyses, some genotypic sequence or structure features could be identified amongst the five JEV genotypes, if any. Such regions would then be selected for phylogenetic analysis. The software used in the analyses is listed in the flow chart of the JEV UTRs data analysis pipeline (Figure 1). The Supplementary Data 2 contains the detailed parameter settings utilized in each software as well as the analysis scripts. The datasets, sequence alignments, and the software used in the current study are available from the corresponding authors upon request.

### The *Japanese Encephalitis Virus* Untranslated Regions Datasets Construction

In order to understand the sequence and structural features of the UTRs of JEV, the nucleotide sequences of the 5′ and 3′UTRs of JEVs were downloaded from GenBank as of January 2020. The complete UTRs sequences with clear background information including location, data, origin (vector or host) were selected, we excluded all missing at least one of the three metadata.

### The *Japanese Encephalitis Virus* Untranslated Regions’ Sequence Analysis

The sequence analysis of the UTRs of the 160 JEV strains was conducted to explore the genetic features among the different JEV genotypes. First, CLUSTALW software (Thompson et al., 1997) was used to align the nucleotide sequences of the UTRs of JEV. The BioEdit and GeneDoc software were then used to perform sequence editing and nucleotide difference analysis in UTRs. The MegAlign incorporated in DNAStar software (Burland, 2000) was used to compute the sequence distance between different strains to generate the similarity matrix. The Mega-X program (Kumar et al., 2018) was used to analyze the base composition of the nucleotide sequences of the JEV UTRs. A scatter plot was then generated with ggplot2 package in RStudio^1^ to show the proportion of bases (A, U, G, C) of each strain in different genotypes. The statistical analyses were conducted using SPSS software.

### The Repeat Sequences Analysis of *Japanese Encephalitis Virus* and Representative Mosquito-Borne *Flaviviruses*

The UTR sequence alignments were generated using the CLUSTALW software. The GenDoc program was subsequently employed to extract the consensus sequences from the alignments. To determine whether there are repetitive sequences located in the UTRs of representative arboviral *Flaviviruses* (WNV, YFV, ZIKV, DENV) were also downloaded from GenBank for analysis. The NovoPro online tool that is based on the k-mer algorithm (Lerat, 2010) was used to search repeat sequences in the viral UTRs sequences. The core number setting of the minimum repeat sequence followed three principles: (1) The repetitive sequence unit should be obtained; (2) The longest repetitive sequence should be obtained; (3) There should be no overlap between two or more repetitive sequence units. Based on the three principles, 5 and 8 were the best minimum repeat sequence core numbers for the 5′ and 3′ UTRs, respectively. The IBS 1.0.3 software (Liu et al., 2015) was used for results visualization.

### Higher-Order Structure Analysis of the Untranslated Regions of *Japanese Encephalitis Virus*

Prediction of the JEV UTR secondary structure was done using the Mfold software v 3.6 (Zuker, 2003). The parameters used included a folding temperature of 37°C, an ionic condition of 1M NaCl with no divalent ions, and a 5% suboptimality. The upper bound of the number of computed folding and the maximum upper bound of the total number of single-stranded bases allowed in a bulge or interior loop were set at 25. The other parameters were set at default, and the initial ΔG was selected as the smallest structure to obtain the Vienna format file. The representative *Flaviviruses’* (WNV and YFV) genome sequences with confirmed UTR secondary structures were used as references to validate the parameter settings. Taken the representative *Flaviviruses* as reference, a 50 nt-length sequence located after the start codon within the ORF that forms secondary structures, which are essential for genome cyclization, was also included in the present analysis. The VARNA program (Darty et al., 2009) was finally used for visualization of the UTR secondary structure.

### The Phylogenetic Analysis Based on the *Japanese Encephalitis Virus* Untranslated Regions

According to results of sequence alignment of 3′UTRs, it is clear that the 3′ variable region (VR) of JEV 3′UTR, which comprises 300 nt, exhibits clear genotype-specific features. Thus, this region was selected to conduct the phylogenetic analysis. Phylogenetic analyses based on the 5′ and 3′UTRs, as well as on the 3′VR sequences, were all performed using both Maximun Likehood (ML) and Neighbor-Joining (NJ) methods within MEGAX software. The best-fit substitution model of each dataset was estimated using ModelFinder (Kalyaanamoorthy et al., 2017) incorporated in the PhyloSuite software (Zhang et al., 2020). In order to verify the consistency of the phylogenetic trees generated using different gene regions, the phylogenetic tree were also generated using ORF, preM and E gene sequences. The topology of the phylogenetic trees based on the different gene datasets were compared using the Robinson–Foulds (RF) metric (Robinson and Foulds, 1981; Smith, 2020) to validate the accuracy of JEV genotyping based on the VR sequences. The ML and NJ trees were constructed with 1000 bootstrap replicates.

## Results

### The *Japanese Encephalitis Virus* Untranslated Regions Datasets Construction

After removing the sequence with insufficient background information and incomplete nucleotides, the sequences of 93 JEV strains were selected, together with the sequences of 67 strains from our lab, to form a final whole-genome sequence dataset containing 160 JEV strains. Then the JEV whole-genome sequence dataset was split into three parts to form the 5′ UTR, ORF, and 3′ UTR gene datasets. The 160 JEV strains are representing the samples isolated from kinds of mosquitoes (*n* = 82)with *Culex tritaeniorhynchus* as the major species (*n* = 46), midges (*n* = 2), bats (*n* = 6), pigs (*n* = 29), horses (*n* = 2), and humans (*n* = 38). The isolation dates ranged from 1935 to 2018. The isolation sites including China (*n* = 121), Japan (*n* = 17), India (*n* = 7), South Korea (*n* = 4), South Korea (*n* = 2), Vietnam (*n* = 1), Laos (*n* = 1), Australia (*n* = 1), Angola (*n* = 1), Indonesia (*n* = 1), Malaysia (*n* = 1), Singapore (*n* = 1), and Philippines (*n* = 1). The geographical distribution extends from latitude 15°S to latitude 45°N. The 160 strains of JEV belongs to five genotypes: GI (*n* = 78), GII (*n* = 1), GIII (*n* = 77), GIV (*n* = 1), and GV (*n* = 3) (detailed information in Supplementary Table 1).

### The Sequence Analyses of the *Japanese Encephalitis Virus* Untranslated Regions

Both of the 5′ and 3′ UTRs sequences were compared and analyzed using CLUSTALW and GenDoc software. It is found that the length of the 5′ UTR region of JEV is 95 nt (only GI is 96 nt). The 5′ UTRs are more conserved compared with the 3′ UTRs. There are nucleotide differences that exist in both the 5′ and 3′ UTRs among the different genotypes (Tables 1–3 and Supplementary Table 1). The 3′UTR ranges in length from 557 to 596 nt, and the nucleotide sequence of the 3′UTRs is more variable than that of the 5′UTRs. There is a 300 nt-length region, starting from the stop codon, that is the most variable and exhibits a clear genotype specificity (Tables 1, 3). In JEV and other arboviral *Flaviviruses*, the 5′UTR initiates with an adenine (A) that is immediately connected with the 5′cap structure (m^7^G_*ppp*_A_*mp*_N) and terminates with the CU. However, we identified that four out of 160 JEV strains were not terminated with CU.

**TABLE 1:** Primary sequence and secondary structure elements analysis of the JEV’s UTRs.

**UTR**	**5′UTR**						**3′UTR**				
**Genotypes**		**I**	**II**	**III**	**IV**	**V**	**I**	**II**	**III**	**IV**	**V**
Primary sequence	Length (nt)	96	95	95	95	95	565–571	570	557–583	584	586–591
	Similarity	90.7–100% (μ = 98.31%)	—	95.9–100% (μ = 99.36%)	—	99–100% (μ = 99.34%)	95.5–100% (μ = 98.71%)	—	88.6–100% (μ = 97.83%)	—	94.9–100% (μ = 96.6%)
	Nucleotide composition	A = 32.38%	A = 31.58%	A = 31.58%	A = 29.47%	A = 32.63%	A = 28.12%	A = 29.3%	A = 29.17%	A = 29.28%	A = 30.72%
		G = 24.00%	G = 24.21%	G = 24.20%	G = 24.21%	G = 24.21%	G = 29.65%	G = 28.95%	G = 29.03%	G = 28.42%	G = 28.51%
		T = 31.14%	T = 32.63%	T = 32.66%	T = 33.68%	T = 31.93%	T = 18.32%	T = 18.25%	T = 18.51%	T = 18.15%	T = 18.83%
		C = 12.50%	C = 11.58%	C = 11.57%	C = 12.63%	C = 11.23%	C = 23.92%	C = 23.51%	C = 23.42%	C = 24.14%	C = 21.95%
		A > T > G > C	T > A > G > C	T > A > G > C	T > A > G > C	A > T > G > C	G > A > C > T	A > G > C > T	A > G > C > T	A > G > C > T	A > G > C > T
	Repeat sequences (number)	5	4	4	3	4	5	4	5	5	4

Secondary structure (Location and length)	SLA	5–71 (67 nt)	5–71 (67 nt)	5–71 (67 nt)	11–73 (63 nt)	5–71 (67 nt)	na	na	na	na	na
	SLB	73–109 (37 nt)	73–108 (36 nt)	73–108 (36 nt)	85–95 (11 nt)	73–108 (36 nt)	na	na	na	na	na
	5′UAR|	79–103 (13 nt)	79–102 (14 nt)	79–102 (15 nt)	79–102 (13 nt)	79–102 (12 nt)	na	na	na	na	na
	UFS| |	73–109 (12 nt)	73–108 (12 nt)	73–108 (12 nt)	—	73–108 (12 nt)	na	na	na	na	na
	cHP	116–135 (20 nt)	115–134 (20 nt)	115–134 (20 nt)	115–134 (20 nt)	115–134 (20 nt)	na	na	na	na	na
	5′DAR	109–113 (5 nt)	108–112 (5 nt)	108–112 (5 nt)	108–112 (5 nt)	108–112 (5 nt)	na	na	na	na	na
	5′cCS	137–147 (11 nt)	136–146 (11 nt)	136–146 (11 nt)	136–146 (11 nt)	136–146 (11 nt)	na	na	na	na	na
Domain1	VVR	na	na	na	na	na	1–48 (48 nt)	1–49 (49 nt)	1–62 (62 nt)	1–62 (62 nt)	1–74 (74 nt)
	xrRNA1	na	na	na	na	na	54–128 (75 nt)	55–129 (75 nt)	68–142 (75 nt)	68–142 (75 nt)	80–154 (75 nt)
	3′vrSL	na	na	na	na	na	136–208 (73 nt)	137–209 (73nt)	150–222 (73 nt)	150–222 (73 nt)	162–234 (73 nt)
	xrRNA2	na	na	na	na	na	123–278 (67 nt)	214–280 (67 nt)	227–293 (67 nt)	228–293 (66 nt)	238–302 (65 nt)
	PK1|	na	na	na	na	na	83–87 107–111 (3 nt)	84–88 108–112 (3 nt)	97–101 121–125 (3 nt)	97–101 121–125 (3 nt)	109–113 133–137 (3 nt)
	PK2	na	na	na	na	na	240–242 261–263 (3 nt)	241–243 262–264 (3 nt)	254–256 275–277 (3nt)	254–256 275–277 (3 nt)	263–265 284–286 (3 nt)
	3′vrCS1	na	na	na	na	na	154–179 (26 nt)	155–180 (26 nt)	168–193 (26 nt)	168–193 (26 nt)	180–205 (26 nt)
Domain2	DB1	na	na	na	na	na	300–370 (71 nt)	301–371 (71 nt)	314–384 (71 nt)	314–384 (71 nt)	323–393 (71 nt)
	DB2	na	na	na	na	na	381–448 (68 nt)	382–449 (68 nt)	395–462 (68 nt)	395–462 (68 nt)	402–469 (68 nt)
	PK3	na	na	na	na	na	322–328 449–455 (7 nt)	323–329 450–456 (7 nt)	336–342 463–469 (7 nt)	336–342 463–469 (7 nt)	345–351 450–456 (7 nt)
	PK4	na	na	na	na	na	403–407 459–463 (5 nt)	404–408 459–463 (5 nt)	417–421 472–475 (5 nt)	417–421 472–476 (5 nt)	424–428 480–484 (5 nt)

	3′dbRCS	na	na	na	na	na	341–367 419–445 (27 nt)	342–368 420–446 (27 nt)	355–381 433–459 (27 nt)	355–381 433–459 (27 nt)	364–390 440–466 (27 nt)
	3′dbCS2	na	na	na	na	na	381–391 (11 nt)	382–392 (11 nt)	395–405 (11 nt)	395–405 (11 nt)	402–412 (11 nt)
	3′dbsHP	na	na	na	na	na	450–464 (15nt)	451–464 (14 nt)	464–477 (14 nt)	464–477 (14 nt)	471–485 (15 nt)
	3′cCS	na	na	na	na	na	460–470 (11 nt)	460–470 (11 nt)	473–483 (11 nt)	473–483 (11 nt)	481–491 (11 nt)
	3′sHP	na	na	na	na	na	473–489 (17 nt)	473–489 (17 nt)	486–502 (17 nt)	486–502 (17 nt)	494–510 (17 nt)
	3′DAR	na	na	na	na	na	473–478 (5 nt)	473–478 (5 nt)	486–491 (5 nt)	486–491 (5 nt)	494–498 (5 nt)
	3′UAR|	na	na	na	na	na	481–504 (13 nt)	481–504 (14 nt)	495–417 (15 nt)	495–417 (13 nt)	502–525 (12 nt)
	3′SL	na	na	na	na	na	490–573 (84 nt)	490–573 (84 nt)	503–586 (84 nt)	503–586 (85 nt)	511–594 (84 nt)

*| : Non-contiguous nucleic acid sequence; | | : non-contiguous complementary nucleic acid sequence; NA: Not available. The results of nucleotide similarity and base composition were all within genotypes. SLA, stem loop A; SLB, stem loop B; UFS, 5′-UAR-flanking stem; 5′UAR, 5′ upstream AUG region; 5′DAR, 5′ downstream AUG region; cHP, capsid-coding region hairpin; 5′cCS, 5′cyclization sequence; VR, variable region; xrRNA, the exoribonuclease XRN1 resistant RNA; PK, pseudoknot; 3′vrCS1, the 3′UTR variable region conserved sequences 1; 3′dbRCS, the 3′UTR dumbbell structure repeated conserved sequences; 3′dbCS2, the 3′UTR dumbbell structure conserved sequences 2; 3′dbsHP, the 3′UTR DB region small hairpin; 3′cCS, 3′cyclization sequence; 3′DAR, 3′ downstream AUG region; 3′UAR, 3′upstream AUG region upstream AUG region; 3′sHP, the 3′UTR short hairpin; 3′SL, the 3′UTR stem loop.*

**TABLE 2:** The nucleotide differences within the 5′UTRs between five JEV genotypes.

**No.**	**Positions of mutation**	**Standard Strain**	**Genotypes**				
		**Nakayama**	**I**	**II**	**III**	**IV**	**V**
1	28	G					A(3/3)
2	37	C		T(1/1)		T(1/1)	
3	44	A				G(1/1)	
4	45	A					G(3/3)
5	50	A				C(1/1)	
6	79	T				C(1/1)	
7	83	G				A(1/1)	
8	92	–	A(76/78)				
9	93	T	C(77/78)	C(1/1)			A(3/3)

*A, adenine; G, guanine; C, cytosine; T, thymine. “–” indicates missing nucleotides. I-V indicate genotype I to genotype V of JEVs. In parentheses, each denominator indicates the total number of the JEV strains of each genotype while the numerators indicate the number of the mutated JEV strains.*

**TABLE 3:** The nucleotide differences within the 3′UTRs between JEV genotypes.

**No.**	**Position of mutation**	**Standard strain**	**Genotypes**					
		**Nakayama**	**I**	**II**	**III**	**IV**	**V**	
							Muar/Tengah	XZ0934
1	1–13	-					AGAACTCTTGAAA	-AAACTTTTGGTA*
2	24	G	–	–		A	A	A
3	25–26	GT	–	AT			TA	TA
4	28	G				A	T	T
5	36	A					G	–*
6	14–17	TGTG	–	–			ACAA	ATGA*
7	27	A					G	G
8	30–31	AA	GG				GT	GC*
9	33	A	T	T	G		A	–*
10	35	A					T	–*
11	37	A	G	G			T	T
12	38	C	T	T		T	T	T
13	39	C		T	T	G	T	T
14	41	–					G	T*
15	45–47	AAA	GTG	GTA				
16	50	A				G	G	G
17	54	A	G	G		G		
18	56	-	G					
19	59	G	A	A		A	A	A
20	61	G				T	A	A
21	62	A					T	T
22	67	G					T	T
23	68–69	TA	–	–	CA	TG	–	–
24	71	G		A		A	A	A
25	72	C					G	A*
26	75	A	G					
27	76	T					G	G
28	78	G					A	G*
29	88–89	AG					GA	GA
30	91–93	AAA				GTT	GCG	GTA*
31	96–97	CT					TC	TC
32	108–109	TA					AG	TG*
33	113	G	A				A	A
34	133	T					C	C
35	139	T					C	C
36	172	A	G			G	C	C
37	179	G	G	G	G	G	A	A
38	180	A	G				G	G
39	207	T	C			A	A	A
40	219	C					T	T
41	222	T	C	C				
42	226	A				T	G	G
43	231	A		G		G	G	G
44	238–240	AAC					G-T	G-T
45	251–252	AA					G-	G-
46	253	A	T			T	–	–
47	254–256	TTT	TCT		TAT	CTT	TAA	CAA*
48	265	C					T	T
49	271	G				A	A	A
50	279	G					A	A
51	285–286	CG				TA	TA	TA
52	294	A					G	G
53	302	T					C	C
54	307	T					A	A
55	309	C	A	A		A	A	A
56	318–320	TTG					AAA	ATG*
57	338	G					A	A
58	343	G					A	A
59	348	G	A					
60	360	C					T	T
61	361	G	T					
62	365	T					C	C
63	400–401	AC					–	–
64	404	C					G	G
65	407	C	T	T			G	A*
66	419	G					A	A
67	421	C					T	T
68	423–424	CC				CT	TT	TT
69	438	G				A	A	A
70	440–441	AG		AA			GA	GA
71	487	-	A				A	A
72	508	A					T	T
73	515	G				A	A	A
74	528	C					A	A
75	550	T					A	A

*A, adenine; G, guanine; C, cytosine; and T, thymine. “–” indicates missing nucleotides.*

I–V indicate genotype I to genotype V of JEVs. * Indicates in this site the mutation were equal within the strain XZ0934, Muar and Tengah.

### The Nucleotide Similarity and Composition of the *Japanese Encephalitis Virus* Untranslated Regions

**FIGURE 2:**
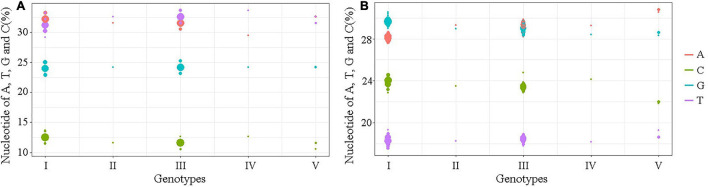
The A, T, G, and C contents of the UTRs of different JEV genotypes. **(A)** The nucleotide contents of the 5′UTRs. **(B)** The nucleotide contents of the 3′UTRs. The abscissa indicates JEV genotypes and the ordinate represents the percentage of nucleotide contents. The red bar identifies adenine (A), green identifies cytosine (C), blue identifies guanine (G) and violet identifies thymine (T).

The nucleotide similarity analysis for the 5′ and 3′UTRs of 160 JEV strains isolated from different vectors, hosts and locations during 1938–2018 revealed that the 5′UTRs exhibit a nucleotide similarity ranges from 84.5 to 100%, with an average similarity of μ = 97.62. The nucleotide similarity of GI and GIII are 90.7–100% and 95.9–100%, respectively. The 3′UTRs exhibit nucleotide similarity ranges from 79.3% to 100%, with a μ = 94.47%. The nucleotide similarity of GI and GIII are 95.5–100% and 88.6–100%, respectively. The detailed similarity of the 5′ and 3′UTRs for each JEV genotype are presented (Tables 1, 4). The JEV 5′UTRs are rich in AT (A + T content reaches 62%), and the 3′UTRs are rich in GC dinucleotides (A + T content is 47%) (Figure 2 and Table 1).

**TABLE 4:** The average nucleotide similarity of the JEVs’ UTRs.

	**Between different genotypes (%)**					**Within each genotype (%)**	
	**I**	**II**	**III**	**IV**	**V**	**5′UTR**	**3′UTR**
I	*	97.06	96.75	90.93	94.72	98.31	98.71
II	94.48	*	97.63	93.80	96.23	99.36	97.83
III	91.88	92.69	*	93.55	96.27	100	100
IV	86.45	87.40	89.44	*	91.07	100	100
V	81.50	81.67	82.25	80.70	*	99.34	96.6

*The average nucleotide similarity of 5′UTR is shown in the upper right corner and the value of 3′UTR is in the lower left corner.*

* indicates identical.

### The Repeat Sequences in the *Japanese Encephalitis Virus* Untranslated Regions

**FIGURE 3:**
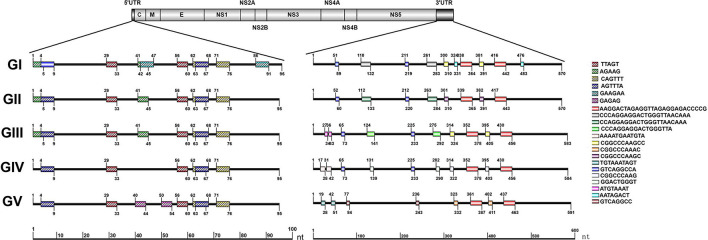
Schematic localization of the repeat sequences in UTRs of different genotypes of JEV. Different color boxes indicate different repeat sequence units. The start and end positions of the repeat sequence unit is labeled upward and downward lines, respectively.

The repeat sequences in the JEV UTRs were analyzed using the NovoPro online tool and IBS 1.0.3 software. We found that both the JEV 5′ and 3′UTRs consist of different numbers of repeat sequence elements. The numbers, locations and nucleotide sequence composition of the repeat units of the 5′ and 3′UTRs are different among the five JEV genotypes. The detailed information is shown in Figure 3 and Table 1.

### The Repeat Sequences in Representative Mosquito-Borne *Flaviviruses*

**FIGURE 4:**
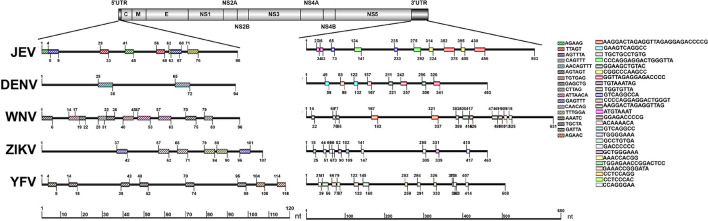
Schematic localization of the repeat sequences in UTRs of medically important *Flaviviruses*. Different color boxes indicate different repeat sequence units. The start and end positions of the repeat sequence unit is labeled upward and downward lines, respectively.

The repeat sequences identification in representative mosquito-borne *Flaviviruses* including WNV, YFV, ZIKV, and DENV were conducted using the NovoPro online tool and IBS 1.0.3 software. The results showed that the repeat sequence elements exist in the 5′ and 3′UTRs of all the analyzed arboviral *Flaviviruses.* There are clearly differences in the length, number, position, and nucleotide sequence composition among the different arboviral *Flaviviruses*. The detailed information is shown in Figure 4.

### The Secondary Structures and Important Functional Sequences in the *Japanese Encephalitis Virus* 5′ Termini

**FIGURE 5:**
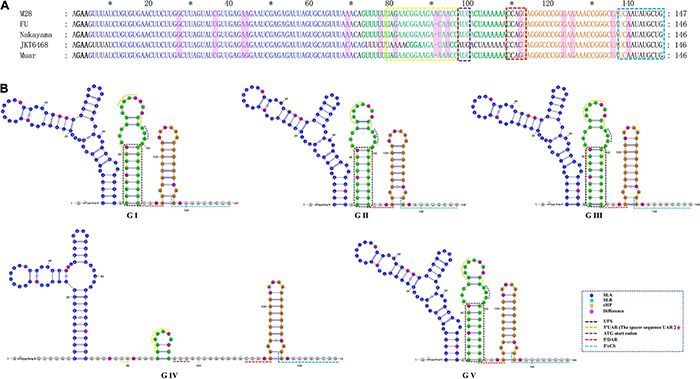
The primary and higher-order structures of the 5′ UTRs of JEV. **(A)** The primary sequences of the JEV 5′ UTRs. **(B)** The higher order structures of the JEV 5′ UTRs. Differently colored nucleotide bases signify different *cis*-acting elements or significant units: bold black, 5′ Cap (m^7^G_*ppp*_A_*mp*_A); blue, the stem-loop A (SLA); green, the stem-loop B (SLB); yellow box, the upstream AUG region (5′ UAR); blue box, the ATG start codon; bold green, the 5′ UAR-flanking stem (UFS); orange, the capsid-coding region hairpin element (cHP); red box, the 5′ downstream AUG region (5′ DAR); aquamarine, 5′ cyclization sequence (5′cCS). The mutation sites are colored magenta. The mentioned units are colored similarly in the higher order structures. Black dashed box, yellow dashed lines and red dashed lines, respectively, denote the UFS, 5′ URA, and 5′ DAR, which were overlapped with other structures and sequences. The red pentagram represents the sequence motif with unique JEV feature.

As shown in Figure 5 and Table 1, the 5′UTRs consist of two stem-loop structures termed SLA and SLB. Within the SLB lies the UAR and the UFS. There are three elements sited after the ATG start codon within the capsid-coding region, termed the DAR, the cHP and the 5′cCS. Except for GIV, the secondary structures of the JEV 5′UTR are conserved among the five JEV genotypes. The 5′UTRs start with an adenine (A) connecting to the 5′ cap structure (m^7^G_*ppp*_A_*mp*_A), directly linked to the SLA. SLA has a conserved structure with a length of about 70 nucleotides. GIV has a unique secondary structure in that instead of a direct connection between the 5′ cap and SLA, a U-rich sequence is inserted. Furthermore, the morphology of SLA of GIV differs from those of other genotypes.

SLB sits closest to the SLA, and contains UFS and 5′UAR. The SLB starting sequence (5′-GUUUUU-3′) and the sequence within the coding region (4 nucleotides behind the start codon, 5′-AAAAAC-3′) are reversely complementary, which form a stem of 6 base pairs in length, termed the 5′UAR-flanking stem (UFS) (Figure 5). An 11 nt-length bulge structure is connected to the JEV UFS structure (stem), which includes both the 6 nt-length 5′UTR sequence and the 5 nt-length sequence from the start codon of the capsid protein-coding sequence (5′-ATGAC-3′). Among all the medically important arboviral *Flaviviruses* (DENV 1-4, YFV, ZIKA, WNV, TBEV), only DENV 4 and ZIKV have this bulge structure, but it lacks an ATG start codon. This structural feature is unique in the JEV 5′ termini. The UAR sequence is located within the top loop of SLB, and the size and nucleotide composition of the top loop structures are different among the five JEV genotypes.

There is a clear difference that exists in the loop structure in JEV GI, due to the adenine insertion at position 92, as well as a mutation (93 T > C), resulting in the top loop structure change of SLB. The UARs of JEV are not continuous sequences but consists of four interspaced sequences (Denoted by yellow dashed lines in Figure 5). When compared to other mosquito-borne *Flaviviruses* (WNV, YFV, ZIKV, DENV), which have continuous UARs, the feature of JEV sequence is unique. We defined the JEV’s UARs as spacer sequence UAR. The 5’ DAR is located downstream of SLB, within the linker of SLB and cHP (Denoted by red dashed lines in Figure 5). It is conserved among JEV genotypes. The cHP contains a CG-rich stem and an A-rich top loop. The cHP’s loop structure of GI is larger than GIII. The 5′ cCS is an 11nt long conserved sequence motif within JEV genotypes and is located downstream the cHP.(5′ cCS:5′-TCA ATATGCTG-3′). Except for JEV GIV, the cCS sequences of the other four JEV genotypes differ only in the position of the starting codon. The JEV GIV’s cCS sequence is 5′-TCTATATGCTG-3′, located 39 nt downstream of the ATG start site. There is a mutation (A > T) at the third position within the cCS sequence (Figure 5).

### The Secondary Structures and Important Functional Sequences in the *Japanese Encephalitis Virus* 3’UTR

**FIGURE 6:**
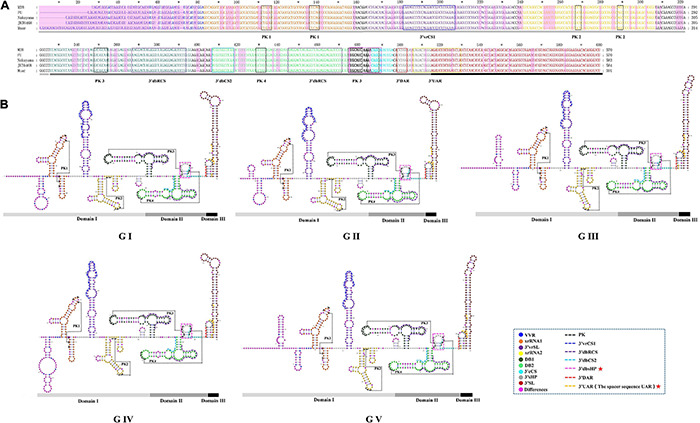
The primary and higher order structures of the 3′UTRs of JEV. **(A)** The primary sequences of the JEV 3′UTRs. **(B)** The higher order structures of the JEV 3′UTRs. Differently colored nucleotide bases signify different structures, *cis*-acting elements or significant sequence motifs: blue, the very variable region (VVR); orange, the exoribonuclease resistant RNA (xrRNA1); purple, the 3′UTR variable region stem loop (3′vrSL); light yellow, the exoribonuclease resistant RNA (xrRNA2); dark green, dumbbell structure 1 (DB1); light green, dumbbell structure 2 (DB2); bold (black and aquamarine), the 3′UTR DB region small hairpin (3′dbsHP); aquamarine, 3′cyclization sequence (3′cCS); gray, the 3′UTR short hairpin (3′sHP); brick red, the 3′UTR stem loop (3′SL). The *cis*-acting elements or sequence motifs which were overlapped with other structures were labeled with different colored dashed line box: black, pseudoknot PK; blue, the 3′UTR variable region conserved sequences 1 (3′vrCS1); purple, the 3′UTR dumbbell structure repeated conserved sequences (3′dbRCS); light blue, the 3′UTR dumbbell structure conserved sequences 2 (3′dbCS2); bright pink, the 3′UTR DB region small hairpin (3′dbsHP); red, the 3′downstream AUG region (3′DAR); yellow, the 3′ upstream AUG region (3′UAR). The magenta blocks represent the differences within five JEV genotypes. The Domain I, II, and III are indicated with light gray, dark gray and black bars, respectively. The mentioned units are labeled similarly in the higher order structures. The red pentagram represents the JEV specific structure or sequence motifs.

We found the 3′UTR of JEV consists of three domains, termed domain I, II, III (Figure 6). Domain I comprised approximately 300 nt, which is immediately downstream of the stop codon, and is highly variable and termed the variable region (VR). The VR contains a very high variable region (VVR) and three stem-loop stem structures. The VVR ranges from 48 to 74 nt in length and is enriched in AU dinucleotides. The secondary structures of the VVR of the different JEV genotypes are significantly different (Figure 6). There are two SL structures that are resistant to host 5′-3′exoribonuclease (XRN1) activity, termed xrRNA1 and xrRNA2, which are conserved in the secondary structure of the five JEV genotypes. In xrRNA1, a three-nucleotide spacer sequence (5′-C-G-G-3′) located in the long-arm top loop pairs with a reverse complementary spacer sequence (5′-C-C-G-3′) that lies in the stem structure to form the pseudoknot (PK) PK1 (Figure 6, labeled with black dashed line). In xrRNA2, a three-nucleotide sequence (5′-GCU-3′) located in the long-arm top loop pairs with a 3-nucleotide sequence (5′-AGC-3′) that lies in the linear sequences between the SL structures to form PK2. The xrRNA1 and xrRNA2 structures of JEV are separated by a long SL structure, which is 73 nt in length. The structure is moderately conserved in the different JEV genotypes, and we named it the 3′UTR variable region stem loop (3′vrSL). There is a 25 nt-length conserved sequence, termed the 3′UTR variable region conserved sequences 1(3′vrCSl), which lies at the top loop of the 3′vrSL. A short SL structure with a length of approximately 11 nt is located after the xrRNA2 structure. The stem structures of the short SL structure are highly conserved in the five JEV genotypes, whereas the loop sequences are divergent. For genotypes I-III, the loop sequences are conserved (5′-GUUGA-3′), and for GIV and GV, the sequences are 5′-CAUGA-3′ and 5′-GAAAA-3′, respectively (Figure 6). Domain II consists of two dumbbell structures (DB1, DB2) and a 15 nt-length small hairpin structure. Theses structures are highly conserved among the five JEV genotypes. After searching the 3′UTRs of other mosquito-borne *Flavivirus* (WNV, YFV, ZIKV, and DENV), it is found that the 15 nt-length small hairpin structure could only be identified in the DB region in 3′UTRs of JEV. Thus, we named the it the 3′UTR DB region small hairpin (3′dbsHP). The long-arm top loop of DB1 (5′-GAUGCAA-3′) pairs with a reverse complementary sequence (5′-UUGCAUC-3′), which lies in the 3′dbsHP, to form PK3. The long-arm top loop of DB2 (5′-GCUGU-3′) pairs with a reverse complementary sequence (5′-ACAGC-3′) in the 3′dbsHP to form PK4 (Figure 6). The two PKs serve to further stabilize the DB structures. Besides the PKs located in the 3′dbsHP, partial sequences of 3′cCS sequence (5′-**CAG CA**U AUU GA-3′) overlaps the 3′dbsHP (Figure 6, bright pink dashed box). In the internal loop and the short arm structure of both DB1 and DB2, there are 27 nt-length repeated conserved sequences (-AAGGACUAGAGGUUUAGAGGA GACCCG-), designated the 3′UTR dumbbell structure repeated conserved sequences (3′dbRCS).

Domain III is considered to be highly conserved across the *Flaviviruses* and is also known as a highly conserved region (HCR). However, we found that there are structural differences that exist in the JEV genotypes. Domain III is approximately 100 nt in length, consisting of a hairpin named the 3′UTR short hairpin (3′sHP) and a long terminal SL structure termed the 3′UTR stem loop (3′SL). The 3′SL structures are almost identical among GI to GIII. However, the shapes of the 3′SL structures of GIV and GV differ from those of GI to GIII (Figure 6). The sequence (5′-CCUGG-3 ′) that lies in the stem of 3′sHP is 3′DAR. In the 3′SL structure, the 3′UAR, which consist of four discrete spacer sequences, that complement the 5′ UAR (Figure 6).

### Sequence Feature Information-Guided *Japanese Encephalitis Virus* Genome Typing

**FIGURE 7:**
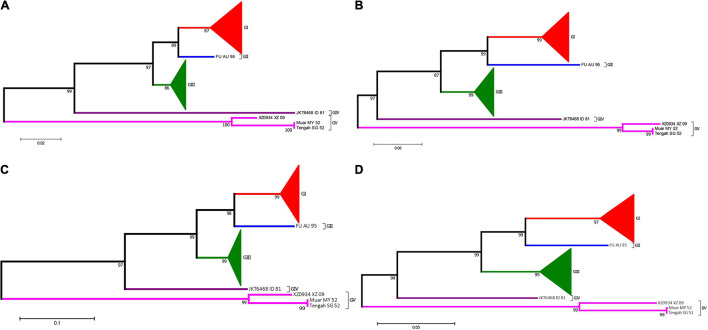
Phylogenetic analysis of JEV. **(A)** The phylogenetic tree based on the sequences (1–300 nt) of domain I (VR) within the 3′UTRs of JEV. **(B)** The phylogenetic tree based on the PrM gene of JEVs. **(C)** The phylogenetic tree based on the E gene of JEVs. **(D)** The phylogenetic tree based on the ORF gene of JEVs. Red, blue, green, purple and violet were used to mark genotype I, II, III, IV, and V of JEV, respectively. The triangles were used to condense strains of the same genotypes.

Prior to generate the NJ phylogenetic tree, the average evolutionary divergence over all sequence pairs was computed. The average divergence values for 5′UTR, 3′UTR, and 3′VR are 0.09 ± 0.01, 0.07 ± 0.01, and 0.04 ± 0.01, respectively. The results proves the datasets are suitable for generating distance based phylogenetic trees. The ModelFinder program was used to select the best-fit nucleotide substitution model based on the Bayesian information criterion (BIC) before generating the ML tree. The best-fit models for the 3′VR, PrM, E and the ORF gene datasets were GTR + G4, GTR + G4, GTR + I + G4, GTR + F + I + G4, respectively. The phylogenetic analyses showed that the JEV isolates can be clustered into five genotypes based on the JEV 3′UTR′s VR gene sequences using both ML and NJ methods. The normalized RF values of the 3′VR tree vs. the ORF, E, and preE trees were 0.29, 0.31, and 0.34, respectively. Further analysis revealed that the main branches of the different phylogenetic trees were identical (Figure 7).

## Discussion

This study provides comprehensive and detailed information of the sequence features and the exact sequence composition, position and distribution of the *Flavivirus*-conserved *cis*-acting elements in the JEV UTRs. The 5′ and 3′ terminal structures of JEV share the general characteristics of *Flaviviruses*, the reported structures and *cis*-acting elements of other *Flaviviruses* are all existed in the UTRs of JEV. The structure and *cis*-acting elements’ sequence comparison with other mosquito-borne *Flaviviruses* (WNV, YFV, ZIKV, DENV) demonstrated the existence of a JEV-specific structure and a sequence motif with a unique feature of JEV UTRs. (i) The 3′ dbsHP, which is a small hair-pin structure located in the DB region within the 3′ UTR. The 3′ dbHP is highly conserved among five JEV genotypes and contains three functional important sequences (PK3, PK4, and 3′ cCS) that involved in DB structure maintaining and viral genome cyclization. (ii) The spacer sequence UARs. The UARs of JEV consist of four discrete spacer sequences whereas the UARs of other mosquito-borne *Flaviviruses* (WNV, YFV, ZIKV, DENV) are continuous sequences. During viral RNA syntheses, the 5′UAR hybridizes with 3′UAR to form a complementary pairing structure that aids viral genome cyclization. The molecular dynamics and the function of the segmented UARs in JEV genome cyclization remain unclear. Given the importance of JEV, it is worth exploring the molecular functions of the unique structure and *cis*-acting element existent in the UTRs.

Our findings demonstrated that repeated sequences are found not only in the UTR of JEV but also in those of the other medically important mosquito-borne *Flaviviruses*, including WNV, YFV, ZIKV, and DENV. However, these repeated sequences differ in nucleotide composition, length and position among the different viruses. These results indicate that the UTRs of *Flaviviruses* contain repeated sequences, but these sequences are not consistently conserved across species. Although repeated sequences are present in the UTRs of GI–V JEV, their lengths, distances between sequences, distributions in the UTR, distances between the repeated sequences and the initiation (termination) codons differ among the genotypes. Despite differences in host origin, regional distribution and isolation time, the length and position of the repeated sequences are highly conserved within the same JEV genotype. Therefore, these repeated sequences are important molecular markers for distinguishing JEV genotypes. The interaction between the conserved regions of the viral sequence and binding proteins is a long-term evolutionary interaction between the virus and the host cell. Even minor mutations in the nucleotide sequence can affect the binding affinity of the interacting proteins (Jin, 2001). *In vitro* experiments have demonstrated that removal of all repeat sequences from *Alphaviruses* maintains their viabilities but reduces their titers (Jin, 2001). However, the decrease in virus titer significantly varied among the different host cells (Zhai et al., 2008). This suggests that the repeated sequences can directly affect the replication efficiency of the virus and play varying roles in different host cells, demonstrating a significant host specificity. The latest research reveals that the repeated conserved sequences in the 3’UTR are involved in subgenomic flaviviral RNA production and viral virulence, indicating the importance of the repeated sequences for *Flaviviruses* (Zhang et al., 2020). JEV has the longest repeated sequences compared with other *Flaviviruses*, indicating that the use of long repeated sequences for completing the virus lifecycle is unique to JEV. These repeated sequences might play an important role in the successful viral replication and transmission. Nonetheless, further studies will be required to confirm the influence of these repeated sequences on viral biological functions.

In the present study, we find that the 3′UTRs are more divergent than the 5′UTRs in both the primary sequence and the higher-order structures of JEV. The JEV 3′UTR consists of three domains. Domain I is considered to be the most variable region within the 3′UTRs among all the arthropod-borne *Flaviviruses*, and thus this region has been termed the variable region (VR) (Proutski et al., 1997; Ng et al., 2017). In our study, it was also demonstrated that domain I is the most variable region within JEV and that this 300 nt-length region exhibits obvious genotype-specific features.

Currently, JEV can be classified into five genotypes (GI to GV), and the distribution, host range and pathogenesis among the five genotypes are quite different (Wang and Liang, 2015; Gao et al., 2019). In particular, JEV has already spread from its traditional endemic region in Asia to Europe (Platonov et al., 2012; Ravanini et al., 2012), and JEV-infected patients have even been identified in Africa (Simon-Loriere et al., 2017). Therefore, identification of the JEV genotype has an important significance for the prevention and treatment of JE. The current methods for JEV genotyping are all based on the protein-coding region sequences, such as the E or the PrM gene (Chen et al., 1990, 1992; Solomon et al., 2003; Wang et al., 2007). Our study demonstrated that the JEV 3′UTR VR region exhibits a strong genotype-specific and that this specificity does not change with the isolation time, distribution or the host (vector). Therefore, this 300 nt-long nucleotide sequence is ideal for JEV genotyping. The phylogenetic analysis results, based on the complete ORF genome sequence (10,300 nt), the E gene (1500 nt), the PrM gene (500 nt) and the 3′UTR conserved sequence (300 nt) using different phylogenetic algorithms, generated consistent results (Figure 6), which further suggests that the VR region sequence within the 3′UTR is an ideal sequence marker for JEV genotyping. Whether the VR region sequences of other *Flaviviruses* also exhibit genotype-specific features and could have been used for genome typing needs further evaluation.

Our study compared the sequence and predicted structural differences in the 5′ and 3′ UTRs of 160 JEV strains isolated over an 80-year period. GI and GIII are the most widespread genotypes of JEV and cause human viral encephalitis and animal diseases (Zheng et al., 2012). All the previously reported GI and GIII strains were included and analyzed to systematically compare them and identify any nucleotide changes attributed to time, host, vector, and geographical distribution. We revealed nucleotide differences in the 5′ and 3′ UTR associated with genotype but not isolation time, host, vector, or geographical distribution. The imbalance in sequence information across the JEV genotypes in the JEV sequence dataset is because only a few JEV strains belonging to GII, GIV, and GV (Williams et al., 2000; Li et al., 2011; Woo et al., 2020) have been isolated in nature. Enhanced surveillance of JEV in nature to obtain sufficient genomic sequence information of different JEV genotypes to improve the understanding of its comprehensive genetic characteristics is highly recommended.

## Data Availability Statement

The original contributions presented in the study are included in the article/Supplementary Material, further inquiries can be directed to the corresponding author/s.

## Author Contributions

HL and GL carried out the study design and contributed to the revision of the manuscript. HL, JZ, and YN performed the experiment and data analysis. GL and HL wrote the manuscript. All authors read and approved the final manuscript.

## Conflict of Interest

The authors declare that the research was conducted in the absence of any commercial or financial relationships that could be construed as a potential conflict of interest.

## Publisher’s Note

All claims expressed in this article are solely those of the authors and do not necessarily represent those of their affiliated organizations, or those of the publisher, the editors and the reviewers. Any product that may be evaluated in this article, or claim that may be made by its manufacturer, is not guaranteed or endorsed by the publisher.

## Footnotes

1
https://rstudio.com
