# Supplementary material for: The 5′ and 3′ Untranslated Regions of the Japanese Encephalitis Virus (JEV): Molecular Genetics and Higher Order Structures
Source: Front Microbiol. 2021 Oct 28;12:730045. doi: 10.3389/fmicb.2021.730045 (PMC8581615; doi:10.3389/fmicb.2021.730045)
Supplement: Supplementary file 1 [file Data_Sheet_1.PDF]

Supplementary Table 1 The background information of the JEV isolates analyzed in this study

NO.	Strain	Host	Date	Location	Genotype	Accession No.
1	M28	*Culex pseudovishnui*	1977	China:Yunnan Province	I	JF706279.1#
2	YN79Bao83	*Culex tritaeniorhynchus*	1979	China:Yunnan Province	I	JN381851.1#
3	YN82BN8219	Mosquito	1982	China:Yunnan Province	I	JN381834.1#
4	BN82215	*Culex annulus mosquitoes*	1982	China: Yunnan Province	I	KT957423.1
5	YN83-Meng83-54	Lasiohelea taiwana Shiraki	1983	China:Yunnan Province	I	JF706282.1#
6	90VN70	Homo sapiens	1990	Viet Nam	I	HM228921.1
7	K94P05	mosquito	1994	Korea	I	AF045551.2
8	Ishikawa	*Culex tritaeniorhynchus*	1994	Japan	I	AB051292.1
9	SH80	*Culex tritaeniorhynchus*	2001	China:Shanghai Province	I	JN381848.1#
10	SH53	*Culex tritaeniorhynchus*	2001	China:Shanghai Province	I	JN381850.1#
11	JEV/sw/Mie/41/2002	swine	2002	Japan: Mie	I	AB241119.1
12	LN02-102	*Culex modestus*	2002	China:Liaoning Province	I	JF706278.1#
13	KV1899	swine	2003	Korea	I	AY316157.1
14	JEV/eq/Tottori/2003	Equus caballus	2003	Japan:Tottori	I	AB594829.1
15	SH03105	*Culex tritaeniorhynchus*	2003	China:Shanghai Province	I	JN381846.1#
16	SH03103	*Culex tritaeniorhynchus*	2003	China:Shanghai Province	I	JN381847.1#
17	HN0411	*Culex*	2004	China:Henan Province	I	JN381831.1#
18	SC04-17	*Culex tritaeniorhynchus*	2004	China:Sichuan province	I	GU187972.1
19	SC0412	*Culex*	2004	China:Sichuan province	I	JN381839.1#
20	SC0415	*Culex tritaeniorhynchus*	2004	China:Sichuan province	I	JN381838.1#

Supplementary Table 1 The background information of the JEV isolates analyzed in this study (Continued 2)

21	JEV/sw/Mie/40/2004	swine serum	2004	Japan: Mie	I	AB241118.1
22	JEV/Sw/Mie-34/2004	Sus scrofa domesticus	2004	Japan: Mie	I	AB698909.1
23	HN0421	*Culex*	2004	China:Henan Province	I	JN381841.1#
24	K05GS	*Culex tritaeniorhynchus*	2005	South Korea: Gunsan-si	I	KR908702.1
25	YN05124	*Culex tritaeniorhynchus*	2005	China:Yunnan Province	I	JF706281.1#
26	YN05155	*Culex tritaeniorhynchus*	2005	China:Yunnan Province	I	JN381852.1#
27	GX0523-445	*Culex tritaeniorhynchus*	2005	China:Guangxi Province	I	JN381832.1#
28	GX0519	*Culex tritaeniorhynchus*	2005	China:Guangxi Province	I	JN381835.1#
29	JEV/Sw/Mie/84/2005	Sus scrofa domesticus	2005	Japan: Mie	I	AB698906.1
30	JEV/Sw-Tokyo/602/2005	Sus scrofa domesticus	2005	Japan: Tokyo	I	AB698908.1
31	JEV/Sw/Tokyo/373/2005	Sus scrofa domesticus	2005	Japan: Tokyo	I	AB698907.1
32	BL06-54	*Culex tritaeniorhynchus*	2006	China:Guangxi Province	I	JF706271.1#
33	BL06-50	*Culex tritaeniorhynchus*	2006	China:Guangxi Province	I	JF706270.1#
34	HN06129	*Armigeres*	2006	China:Henan Province	I	JF706277.1#
35	HN0621	*Culex*	2006	China:Henan Province	I	JN381830.1#
36	HN0626	*Culex*	2006	China:Henan Province	I	JN381837.1#
37	YN0623	*Culex tritaeniorhynchus*	2006	China:Yunnan Province	I	JN381836.1#
38	JEV/Sw/Mie/51/2006	Sus scrofa domesticus	2006	Japan: Mie	I	AB698905.1
39	SH17M-07	mosquito	2007	China:Shanghai Province	I	EU429297.1
40	XJP613	*Culex tritaeniorhynchus*	2007	China:Henan Province	I	EU693899.1
41	YNTC07172	*Culex tritaeniorhynchus*	2007	China: Yunnan Province	I	KT957419.1

Supplementary Table 1 The background information of the JEV isolates analyzed in this study (Continued 2)

42	YNTC07018	*Culex tritaeniorhynchus*	2007	China: Yunnan Province	I	KT957420.1
43	HEN0701	swine brain	2007	China:HeNan Province	I	FJ495189.1
44	131V	Homo sapiens	2007	China:Guangxi Province	I	GU205163.1
45	GS07TS11	*Culex tritaeniorhynchus*	2007	China:Gansu Province	I	JN381843.1#
46	LN0716	*Culex tritaeniorhynchus*	2007	China:Liaoning Province	I	JN381849.1#
47	JX61	pig	2008	China:Henan Province	I	GU556217.1
48	GSBY0801	*Culex tritaeniorhynchus*	2008	China:Gansu Province	I	JF706274.1#
49	GSBY0810	*Culex tritaeniorhynchus*	2008	China:Gansu Province	I	JN381840.1#
50	GSBY0861	*Culex tritaeniorhynchus*	2008	China:Gansu Province	I	JN381833.1#
51	GSBY0827	*Culex tritaeniorhynchus*	2008	China:Gansu Province	I	JN381845.1#
52	GSBY0804	*Culex tritaeniorhynchus*	2008	China:Gansu Province	I	JN381844.1#
53	GZ56	Homo sapiens	2008	China:Guizhou Province	I	HM366552.1#
54	GSBY0816	*Culex tritaeniorhynchus*	2008	China:Gansu Province	I	JN381842.1#
55	YL2009-4	mosquito	2009	Taiwan Province	I	JF499789.1
56	TC2009-3	mosquito	2009	Taiwan Province	I	JF499788.1
57	SD0810	*Culex tritaeniorhynchus*	2009	China:Shandong Province	I	JF706286.1#
58	SX09S-01	swine	2009	China:Shanxi Province	I	HQ893545.1
59	YN09M57	*Culex tritaeniorhynchus*	2009	China: Yunnan Province	I	KT229574.1
60	JEV/CNS769/Laos/2009	Homo sapiens	2009	Laos	I	KC196115.1
61	TC2009-1	mosquito	2009	Taiwan Province	I	JF499790.1
62	YN0967	*Culex tritaeniorhynchus*	2009	China:Yunnan Province	I	JF706268.1#

Supplementary Table 1 The background information of the JEV isolates analyzed in this study(Continued 3)

63	YN0911	*Culex tritaeniorhynchus*	2009	China:Yunnan Province	I	JF706267.1#
64	XZ0938	mosquito	2009	China:Xizang Province	I	HQ652538.1#
65	ZJ10-10	*Culex tritaeniorhynchus*	2010	China: Zhejiang Province	I	KY650727.1
66	ZJ10-7	*Culex tritaeniorhynchus*	2010	China: Zhejiang Province	I	KY650726.1
67	DH10M978	*Culex tritaeniorhynchus*	2010	China: Yunnan Province	I	KT229573.1
68	DH10M865	*Culex tritaeniorhynchus*	2010	China: Yunnan Province	I	KT229572.1
69	DHL10M62	*Culex tritaeniorhynchus*	2010	China: Yunnan Province	I	KT229575.1
70	SCCZ	mosquito	2010	China: Sichuan Province	I	KU351667.1
71	HL2010-2	mosquito	2010	Taiwan Province	I	JQ031753.1
72	DH10M585	*Culex tritaeniorhynchus*	2010	China: Yunnan Province	I	KT957421.1
73	SCYA201201	Sus scrofa	2012	China:Sichuan province	I	KM658163.1
74	JEV/MQ/Yamaguchi/2013/2	*Culex tritaeniorhynchus*	2013	Japan: Yamaguchi, Yoshida	I	AB981184.1
75	JEV/MQ/Yamaguchi/2013/1	*Culex tritaeniorhynchus*	2013	Japan: Yamaguchi, Yoshida	I	AB981183.1
76	10S3	pig	2013	China:Henan Province	I	MF542268.1
77	SCMY	swine	2014	China: Sichuan Province	I	KU351668.1
78	JS-1	*Culex tritaeniorhynchus*	2015	China:Jiangsu province	I	KX357114.1
79	FU	Human	1995	Australia	II	AF217620.1
80	SA14-14-2				III	AF315119.1
81	Nakayama	Human brain	1935	Japan	III	EF571853.1
82	Beijing-1	Human brain	1949	China:Beijing Province	III	L48961.1
83	p3	mosquito	1949	China:Beijing Province	III	U47032.1

Supplementary Table 1 The background information of the JEV isolates analyzed in this study(Continued 4)

84	47	CSF	1950s	China:Heilongjiang Province	III	JF706269.1#
85	SA14	mosquito	1954	Shaanxi Province	III	U14163.1
86	YN	CSF	1954	China:Yunnan Province	III	JN381871.1#
87	CZX	CSF	1954	China:Fujian Province	III	JN381865.1#
88	CBH	CSF	1954	China:Fujian Province	III	JN381860.1#
89	ZMT	CSF	1955	China:Fujian Province	III	JF706283.1#
90	ZSZ	CSF	1955	China:Fujian Province	III	JN381862.1#
91	LFM	Human blood	1955	China:Fujian Province	III	JN381863.1#
92	YLG	CSF	1955	China:Fujian Province	III	JF706280.1#
93	CH13	CSF	1957	China:Sichuan Province	III	JN381870.1#
94	LYZ	CSF	1957	China:Fujian Province	III	JN381869.1#
95	HVI	human	1958	Taiwan Province	III	AF098735.1
96	Vellore P20778	Human	1958	India	III	AF080251.1
97	JaGAr 01	mosquito	1959	Japan	III	AF069076.1
98	Ha3	CSF	1960s	China:Heilongjiang Province	III	JN381872.1#
99	GSS	CSF	1960s	China:Beijing Province	III	JF706275.1#
100	Ling	Human brain	1965	Taiwan Province	III	L78128.1
101	TL	human	1965	Taiwan Province	III	AF098737.1
102	TC	human	1965	Taiwan Province	III	AF098736.1
103	Anyang-300	pig	1969	South Korea	III	KT447437.1
104	TLA	CSF	1971	China:Liaoning Province	III	JN381868.1#

Supplementary Table 1 The background information of JEV isolates analyzed in this study (Continued 5)

105	JaTAn1/75	Sus scrofa	1975	Japan: Tokyo	III	AB551990.1
106	GP78	Human	1978	India	III	AF075723.1
107	HYZ	Patient blood	1979	China:Yunnan Province	III	JN381853.1#
108	JaOArS982	mosquito	1982	Japan	III	M18370.1
109	ZJ82-6	*Culex tritaeniorhynchus*	1982	China: Zhejiang Province	III	KY650724.1
110	ZJ83-8	*Culex tritaeniorhynchus*	1983	China: Zhejiang Province	III	KY650725.1
111	RP-9	mosquito	1985	Taiwan Province	III	AF014161.1
112	RP-2ms	mosquito	1985	Taiwan Province	III	AF014160.1
113	B58	Rousettus leschenaulti	1986	China: Yunnan Province	III	FJ185036.1
114	K87P39	mosquito	1987	South Korea	III	AY585242.1
115	SH3	CSF	1987	China:Shanghai Province	III	JN381864.1#
116	K88A071	*Culex tritaeniorhynchus*	1988	South Korea	III	KR908703.1
117	DH107	*Aedes lineatopennis*	1989	China:Yunnan Province	III	JN381873.1#
118	CH1392	*Culex tritaeniorhynchus*	1990	Taiwan Province	III	AF254452.1
119	JaTAn1/90	Sus scrofa	1990	Japan: Tokyo	III	AB551991.1
120	HB49	Rousettus leschenaulti	1990	China:Yunnan Province	III	JF706284.1#
121	HB97	Rousettus leschenaulti	1990	China:Yunnan Province	III	JF706285.1#
122	JaTAn2/91	Sus scrofa	1991	Japan: Tokyo	III	AB551992.1
123	T1P1	*Armigeres subalbatus*	1997	Taiwan Province	III	AF254453.1
124	GB30	Murina aurata	1997	China: Yunnan Province	III	FJ185037.1
125	014178	human blood clot	2001	India: UP, Lakhimpur district	III	EF623987.1

Supplementary Table 1 The background information of JEV isolates analyzed in this study (Continued 6)

126	04940-4	*Culex quinquefasciatus*	2002	India: Maharashtra, Bhandara district	III	EF623989.1
127	Fj0276	Human blood	2002	China:Fujian Province	III	JN381867.1#
128	HLJ02-134	*Genus Culicoides*	2002	China:Heilongjiang Province	III	JF706276.1#
129	Fj02-29	CSF	2002	China:Fujian Province	III	JF706273.1#
130	FJ0394	Human blood	2003	China:Fujian Province	III	JN381858.1#
131	YN98A151	Mosquitoes	2003	China:Yunnan Province	III	JN381861.1#
132	FJ0339	Human blood	2003	China:Fujian Province	III	JN381859.1#
133	JEV/SW/GZ/09/2004	pig	2004	China: Guizhou Province	III	KF297916.1
134	DL0445	*Armigeres subalbatus and Mansonia uniformis*	2004	China:Yunnan Province	III	JN381854.1#
135	DL04-29	*Culex theileri*	2004	China:Yunnan Province	III	JF706272.1#
136	GZ042	Armigeres	2004	China:Guizhou Province	III	JN381857.1#
137	SH045	*Culex tritaeniorhynchus*	2004	China:Shanghai Province	III	JN381866.1#
138	SH0410	*Culex tritaeniorhynchus*	2004	China:Shanghai Province	III	JN381856.1#
139	JH0418	*Culex whitmorei and Anopheles sinensis*	2004	China:Yunnan Province	III	JN381855.1#
140	057434	human blood clot	2005	India: UP, Gorakhpur	III	EF623988.1
141	SH0601	swine	2006	China:Shanghai Province	III	EF543861.1
142	WHe	swine	2006	China:Shanxi Province	III	EF107523.1
143	JEV/sw/GD/2008	pig	2008	China: Guangdong Province	III	KX965684.1
144	HN2	Scotophilus kuhlii	2008	China:Hainan Province	III	JN711459.1
145	JEV/SW/GD/01/2009	pig	2009	China: Guangdong Province	III	KF297915.1
146	YUNNAN0902	Sus scrofa	2009	China: Yunnan Province	III	JQ086763.1

Supplementary Table 1 The background information of JEV isolates analyzed in this study (Continued 7)

147	YUNNAN0901	mosquito	2009	China: Yunnan Province	III	JQ086762.1
148	GD	Myotis pilosus	2009	China:Guangdong Province	III	JN711458.1
149	JEV/eq/India/H225/2009	horse; breed Marwari	2009	India	III	JX131374.1
150	GZ	swine	2010	China: Guizhou Province	III	KC915016.1
151	SC201301	swine	2013	China:SiChuan province	III	KU363309.1
152	JEV/SW/IVRI/395A/2014	swine	2014	India	III	KP164498.2
153	FC792	swine	2016	China:Guangxi Province	III	MF002373.1
154	C17	Homo sapiens	2016	Angola	III	KX945367.1
155	JEV1805M	Homo sapiens	2018	China：Yunnan Province	III	MN639770.1
156	JEV/sw/Mindanao/K4/2018	Sus scrofa	2018	Philippines	III	LC461960.1
157	JKT6468	mosquito	1981	Indonesia	IV	AY184212.1
158	Muar	Homo sapiens	1952	Malaysia	V	HM596272.1
159	Tengah	Homo sapiens	1952	Singapore	V	KM677246.1
160	XZ0934	mosquito	2009	China:Xizang Province	V	JF915894.1

Note: # indicate the UTR sequence information of the JEV strain was sequenced in our lab and first used in this study.

Supplementary data 2. The detailed parameter settings utilized in each software and analysis scripts used in the study.

1. The JEV UTRs datasets construction

In order to understand the sequence and structural features of the UTRs of JEV, the nucleotide sequences of the 5'and 3'UTRs of JEVs were downloaded from GenBank as of January 2020. The complete UTRs sequences with clear background information including location,data,origin were selected, we excluded all missing at least one of the three metadata.

Detailed procedures:

Enter the NCBI database (https://www.ncbi.nlm.nih.gov/) → select the "Nucleotide" option of the "All Databases" drop-down menu → enter the keyword (*Japanese encephalitis virus*) in the search bar and click search Button to search → select the full genome nucleotide sequence of the standard strain (Nakayama) from the search results to enter the detailed information webpage → click the "Run Blast" button on the right → select the "Max target sequences" value under "General Parameters" to be 500, other parameters default → Click the "Blast" button to search → filter the search results.

2. The JEV UTRs' sequence analysis

First,CLUSTALW software was used to align the nucleotide sequences of the UTRs of JEV.The BioEdit and GeneDoc software were then used to perform sequence editing and nucleotide difference analysis in UTRs. The MegAlign incorporated in DNAStar software was used to compute the sequence distance between different strains to generate the similarity matrix.The Mega-X program was used to analyze the base composition of the nucleotide sequences of the JEV UTRs. A scatter plot was then generated with ggplot2 package in RStudio (https://rstudio.com)to show the proportion of bases (A, U, G, C) of each strain in different genotypes.The statistical analyses were conducted using SPSS software.

Detailed parameter settings of each software:

1. CLUSTALW software was used to align nucleotide sequences of JEV 5’ and 3’UTRs;

Detailed procedures and parameters: Run CLUSTALW → click the "File" button, select "Load Sequences", load the sequence→click the "Alignment" button, select "Do Compete Alignment", click "OK" to perform the full sequence alignment → click "File" Button, select "Save Sequences as" to save the comparison result → click "FASTA" and “MSF” file format to save.

2. The BioEdit software was used to edit the JEVs and *flavivirus* sequences;

Detailed procedures and parameters: Run BioEdit → click the "File" button, select "Open", load the alignment sequence → click the strain name twice to edit the strain name → select the "Edit" option of the "Mode" drop-down menu → select ORF, 5'UTR, 3'UTR in the main page respectively → click the "Cut" button of the "Edit" drop-down menu to cut → get the JEVs ORF, 5'UTR and 3 'UTR sequence respectively → click "File", "Save as" to save as .fasta format.

Operation process: 1.CLUSTALX software was used to perform full sequence

alignment of the

3. The GeneDoc software was used to analyze the difference of nucleotide in the UTRs;

Detailed procedures and parameters:Run the GeneDoc program → click the "File" button, select "Open", import multiple sequence alignment results → click the "Project" button to select "Configure", modify the "Points" to 9 → click the "shade" button, the shading level is no shading, the residue display mode is set to difference, the difference mode style is set to difference/top sequence, and other parameters are default → click the "Edit" button to select "Select Blocks for Copy", and click the main panel to select the export Sequence → Click "Edit" and select "Copy Select Blocks to" and then select "RTF File" to export the .rtf file, and use word to open the file.

4. The Megalign program in the DNAStar program was used to analyze the similarity of the nucleotide in the UTR of the JEVs;

Detailed procedures and parameters:Run the Megalign program → click "File", select "Enter Sequences", then select the sequence file, click "Open" and "Done" in turn, import the nucleotide sequence → click "Align", select "By Clustal W Method" parameters → calculate nucleic acid similarity → click the "View" button, select "Alignment Report" to display the similarity results → result output → excel is used to draw table.

5. TBTools was used to draw a heat map;

Detailed procedures and parameters: Double-click to run the TBTools program → click “HeatMap” of “HeatMap Illustrator” in the “Graphics” drop-down menu → enter the Japanese encephalitis virus similarity data in the data column according to the format of the Set Input ID list sample data → click “Start” button to generate a preliminary heat map → click the "Show Control Dialog" button to set the parameters → check the "show value" to show the percentage of similarity → adjust the "width" and "height" at the bottom of the panel to adjust the picture to a proper size → click " Lucky Color" to adjust the color → click "Save Graph" to save the picture.

6. The statistical package IBM SPSS Statistics software (SPSS) program was used to calculate the mean similarity values;

Detailed procedures and parameters: Run the SPSS program → click "File", select "Open", open the Japanese encephalitis virus similarity data set (Excel table) → select the "Analysis" menu, and select the "Average value" of the "Comparison Average" item options → put the "strain name" into the "independent variable list" content, and put the "value" into the "dependent variable list" list → select the statistical indicators to be calculated into the "cell statistics" box on the right, after selecting the statistics you want to calculate, click "Continue" → output results.

7. The Mega-X program was used to analyze the base composition of the nucleotide sequence of the UTRs of the JEV ;

Detailed procedures and parameters:Process and parameters: Run the Mega-X program → click "File", select "Open A File/Session" and click the aligned UTR sequence file → click "Analyze" → "Input Data Options" and select "Nucleotide Sequences", click "OK", then select "NO", click "Nucleotide Composition" in the

"Statistics" drop-down menu → click the save button to calculate.
8. RStudio (https://rstudio.com/) was used to draw a scatter plot;

Detailed procedures and parameters: Open Rstudio → input script (programming), click "Run" to run the script→after generating the picture, click "Export" to save the picture.

The script is as follows:

```
library(ggplot2)
library(dplyr)
packageVersion("dplyr")
tabletext2 <- read.table('E:\\modules\\JEV.csv',sep=',',stringsAsFactors=T,header = TRUE)
tbl_a <- tbl_df(tabletext2)
ggplot(tbl_a, aes(x = genotypes, y = nucleotide, colour = Group)) +
  geom_point(shape = '_',size= 9)
```

9. The SPSS was used to calculate the average of A, U, G and C content in different genotypes.

Detailed procedures and parameters:Run the SPSS program → click "File", select "Open", open the JEV base composition data set (Excel table) → select the "Analysis" menu, and select the "Average value" in the "Comparison Average" item " options. → put the "strain name" into the "independent variable list" content, and put the "value" into the "dependent variable list" list → select the statistical indicators to be calculated into the "cell statistics" box on the right, and after selecting the statistics you want to calculate, click "Continue" → output results.

3. The repeat sequences analysis of JEV and representative mosquito-borne *flaviviruses*

The UTR sequence alignments were generated using the CLUSTALW software. The GenDoc program was subsequently employed to extract the consensus sequences from the alignments. To determine whether there are repetitive sequences located in the UTRs of representative arboviral *flaviviruses*(WNV, YFV, ZIKV, DENV) were also downloaded from GenBank for analysis. The NovoPro online tool that is based on the k-mer algorithm(Lerat, 2010) was used to search repeat sequences in the viral UTRs sequences. The core number setting of the minimum repeat sequence followed three principles: 1) The repetitive sequence unit should be obtained; 2) The longest repetitive sequence should be obtained; 3) There should be no overlap between two or more repetitive sequence units. Based on the three principles, 5 and 8 were the best minimum repeat sequence core numbers for the 5’ and 3’ UTRs, respectively.The IBS 1.0.3 software (Liu et al., 2015) was used for results visualization.

Detailed parameter settings of each software:

1. GeneDoc was used to obtain the consensus sequence of the UTR sequence of different genotypes of Japanese encephalitis virus and *flavivirus*;

Detailed procedures and parameters: Run the GeneDoc program → click the "File" button, select "Open", and import the multiple sequence alignment results → click the "shade" button, the shading level is four-level shading, the residue display mode is set to normal, and the difference mode style is set to the difference/consistent

sequence line, and the other parameters default → click the "Edit" button to select "Select Blocks for Copy", click the main panel to select the sequence to be exported → then click "Edit" to select "Copy Select Blocks to" and then select "RTF File" to export the .rtf file → open the file with word, and analyze the nucleotide sequences of different genotypes to obtain the consensus sequence.

2. The NovoPro online tool was used to predict the repetitive sequence units of the UTRs of Japanese encephalitis virus and *flavivirus*;

Software: The NovoPro online tool (https://www.novopro.cn/tools/repeats-finder.html)

Detailed procedures and parameters: Open the website (https://www.novopro.cn/tools/repeats-finder.html) → enter the sequence that needs to be predicted the repetitive sequence → set the minimum repeat sequence length → click the "submit" button to predict.Parameter (minimum repetitive sequence length) selection principles: i) The repetitive sequence unit should be obtained; ii) The longest repetitive sequence should be obtained; iii) There should be no overlap between two or more repetitive sequence units. Based on the three principles, 5 and 8 were the best minimum repeat sequence core numbers for the 5' and 3' UTRs, respectively.

3. The IBS program was used to visualize the repeat unit sequences of Japanese encephalitis virus and flavivirus.

Detailed procedures and parameters: Double-click to open IBS → click "Enter" to enter the main page → click the "Protein" button below to set the length and color of each strain's nucleotide (5 genotypes in total) → click "OK" → click below" Domain" button to set the position and length of the repeat sequence and the color displayed → click "OK" → repeat the above nucleotide and repeat sequence settings until the repeat sequence pattern diagram of the 5 genotypes is completed → click the "Export Image" button to save picture.

4. Higher-order structure analysis of the UTRs of JEV

Prediction of the JEV UTR secondary structure was done using the Mfold software v 3.6 (Zuker, 2003). The parameters used included a folding temperature of 370C, an ionic condition of 1M NaCl with no divalent ions, and a 5% suboptimality. The upper bound of the number of computed folding and the maximum upper bound of the total number of single-stranded bases allowed in a bulge or interior loop were set at 25. The other parameters were set at default, and the initial ΔG was selected as the smallest structure to obtain the Vienna format file. The representative *flaviviruses* (WNV and YFV) genome sequences with confirmed UTR secondary structures were used as references to validate the parameter settings. Taken the representative *Flaviviruses* (WNV and YFV) as reference, a 50 nt-length sequence located after the start codon within the ORF that forms secondary structures,which are essential for genome cyclization, was also included in the present analysis. The VARNA program (Darty et al., 2009) was finally used for visualization of the UTR secondary structure.

Detailed parameter settings of each software:

1. The Mfold program was use to predict the secondary structure of the UTR of the JEVs;

Detailed procedures and parameters: Open Mfold (http://www.unafold.org/mfold

/applications) → enter the sequence of the UTR of the JEVs, and predict one by one → set the percent suboptimality number to 5, the upper bound on the number of Secondary structure formed by folding was 20, and the upper bound on the total number of single-stranded bases that are allowed in a bulge or interior loop is 25, other parameters are default → prediction completed, select the smallest structure of Initial ΔG → click the "Vienna" button to save the vienna format file.

2. The VARNA program was used to visualize and analyze the prediction results of the secondary structure of the UTR of the JRVs;

Detailed procedures and parameters:Open VARNA→input the sequence and the corresponding vienna format file in "seq:" and "str:" respectively→click "Create" to obtain the secondary structure→right-click the base in the figure and select By Bases" in the "Colors " drop-down menu to color the selected bases → After the color is completed, right-click and select "Export" to save the picture.

3. RNAcomposer (http://rnacomposer.cs.put.poznan.pl/) was used to predict the tertiary structure of the UTR of the JEVs;

Detailed procedures and parameters: Open RNAcomposer → enter the sequence and vienna format sequence in the sequence input column according to the example file → tick "Select secondary structure prediction method", select the "RNAfold" option → click "Compose" to make predictions → after prediction, select pdb File to download and save.

4. PyMol was used to visualize the results of the tertiary structure of the UTR of the JEVs.

Detailed procedures and parameters: Open PyMol → click "File, Open" in turn to load the pdb file of the tertiary structure of the UTR of the JEVs → click the "Display" button, select "Sequence", and display the tertiary structure sequence at the top → select the analysis required Sequence → click "C" on the right side of the image panel to paint → finish painting, click "File" "Save Image" on the main panel to save the picture.

5.The phylogenetic analysis based on the JEV UTRs

Phylogenetic analyses based on the 5' and 3'UTRs, as well as on the 3'VR sequences, were all performed using both Maximun Likehood (ML) and Neighbor-Joining (NJ) methods within MEGAX software.The best-fit substitution model of each dataset was estimated using ModelFinder (Kalyaanamoorthy et al., 2017) incorporated in the PhyloSuit software (Zhang et al., 2020). In order to verify the consistency of the phylogenetic trees generated using different gene regions, the phylogenetic tree were also generated using ORF, preM and E gene sequences.The topology of the phylogenetic trees based on the different gene datasets were compared using the Robinson-Foulds metric (Robinson and Foulds,1981) to validate the accuracy of JEV genotyping.The ML and NJ trees were constructed with 1000 bootstrap replicates.MEGA-X was used to general the phylogenetic trees of each gene dataset.

1) Neighbor-Joining (NJ) method: Prior to generate the NJ phylogenetic tree,the average evolutionary divergence over all sequence pairs was computed to determine

whether the datasets are fit for generating phylogenetic trees using distance based methods. Only the value of average evolutionary divergence above zero and less then one, the dataset is suitable for generating distance based phylogenetic trees.

i) Compute The average evolutionary divergence over all sequence pairs:

Process and parameters: Run the Mega-X program → Overall Mean Distance→ Set the parameters: Variance Estimation Method (Bootstrap method); No. of Bootstrap Replications (1000); Model/Method(Maximum Composite Likelihood model);Rate among sites (Gamma Distribution,G); Gaps/Missing Data Treatment(Pairwise deletion); Pattern among Lineages (Difference)→ OK → View distance result(example:0.04±0.01 for the 3'VR region gene dataset)→ suitable for generating the NJ tree.

ii) Construct the NJ tree.

Run the Mega-X program→select: Construct/Test NJ tree → Set the parameters → Test for Phylogeny (Bootstrap method); No. of Bootstrap Replications (1000); substitution type (Nucleotide); Model (Maximum Composite Likelihood model); Rate among sites (Gamma Distribution, G); Pattern among lineages: Different (Heterogeneous); Gaps/missing Data treatment (Pairwise deletion) → OK

2) Maximum Likelihood (ML) method: The ModelFinder program incorporated in the PhyloSuite software (Zhang et al., 2020) was used to select the best-fit nucleotide substitution model based on the Bayesian information criterion (BIC) before generating the ML tree.

Process and parameters: Run PhyloSuite v1.2.2 → click "phylogeny" select "ModelFinder" → input alignment file → sequence type: DNA → Threads: AUTO → select "Model for suitable phylogenetic analysis software (BEAST1) → select Criterion: BIC → START → Check the summary.txt file to find the results of the best fit model. Then generating the ML tree. Run the Mega-X program → Construct/Test Maximum Likelihood tree → Set the parameters according to the result of the best-fit model. (GTR); Rate among sites (Gamma Distribution, G); No of Discrete Gamma Categories (4); Gaps/missing Data treatment (Use all sites); ML Heuristic Method (NNI); Test for Phylogeny (Bootstrap method); No. of Bootstrap Replications (1000) → Start analysis

3) The topology comparetion of phylogenetic trees. We use the TreeDistance function in the 'TreeDist' R package to calculate the distance of the trees (Smith,et al;2020). The convenience function TreeDistance returns the variation of clustering information between two trees, normalized against the total information content of all splits.

References:

1.https://ms609.github.io/TreeDist/reference/TreeDistance.html

2.Smith, M.R. (2020) . Information theoretic Generalized Robinson-Foulds metrics for comparing phylogenetic trees. Bioinformatics 36, 5007–5013.

3.MacKay D.J.C. (2003). Information Theory, Inference, and Learning Algorithms. Cambridge University Press, Cambridge.
